# SEA Antagonizes the Imatinib-Meditated Inhibitory Effects on T Cell Activation via the TCR Signaling Pathway

**DOI:** 10.1155/2014/682010

**Published:** 2014-01-02

**Authors:** Guanming Wang, Yuhui Yan, Xiaohua Chen, Chen Lin, Yangqiu Li

**Affiliations:** ^1^Department of Microbiology and Immunology, Medical College, Jinan University, Guangzhou 510632, China; ^2^Quanzhou Medical college, Quanzhou 362000, China; ^3^Institute of Hematology, Medical College, Jinan university, Guangzhou 510632, China; ^4^Key Laboratory for Regenerative Medicine of Ministry of Education, Guangzhou 510632, China

## Abstract

The BCR-ABL kinase inhibitor imatinib is highly effective in the treatment of chronic myeloid leukemia (CML). However, long-term imatinib treatment induces immunosuppression, which is mainly due to T cell dysfunction. Imatinib can reduce TCR-triggered T cell activation by inhibiting the phosphorylation of tyrosine kinases such as Lck, ZAP70, LAT, and PLC**γ**1 early in the TCR signaling pathway. The purpose of this study was to investigate whether the superantigen SEA, a potent T cell stimulator, can block the immunosuppressive effects of imatinib on T cells. Our data show that the exposure of primary human T cells and Jurkat cells to SEA for 24 h leads to the upregulation of the Lck and ZAP70 proteins in a dose-dependent manner. T cells treated with SEA prior to TCR binding had increased the tyrosine phosphorylation of Lck, ZAP70, and PLC**γ**1. Pretreatment with SEA prevents the inhibitory effects of imatinib on TCR signaling, which leads to T cell proliferation and IL-2 production. It is conceivable that SEA antagonizes the imatinib-mediated inhibition of T cell activation and proliferation through the TCR signaling pathway.

## 1. Introduction

Imatinib (IM; formerly STI571) is a tyrosine kinase inhibitor that has strong activity against the BCR/ABL tyrosine kinase, a fusion protein that causes the onset of Philadelphia chromosome positive chronic myeloid leukemia (CML) and broad activity against Src-family tyrosine kinases, such as c-KIT and platelet-derived growth factor receptor, which play a role in gastrointestinal stromal tumors. The introduction of IM for the treatment of CML represents an ideal example of molecular targeted therapy in human cancer. It is also conceivable that although imatinib is a successful kinase inhibitor in clinical use, immunosuppression, an imatinib-associated side effect, is inevitable. Accumulating evidence shows that long-term imatinib treatment induces defects in humoral and cell-mediated immunity [1–5].

A new generation of tyrosine kinase targeted drugs, such as dasatinib and nilotinib, are effective in many cases in which disease is resistant to imatinib. These drugs have been reported to inhibit T cell function in vitro, in mouse models [6–13], and in CML patients [14, 15].

Studies have shown that the inhibition of Lck and other kinases involved in T cell signaling by tyrosine kinase inhibitors, including imatinib, dasatinib, and nilotinib, is responsible for the suppression of T cell function. The Src kinase Lck is a key target of these compounds during T cell activation. These findings raise a concern for potential T cell inhibition in patients taking tyrosine kinase inhibitors. While targeted therapy could induce CML remission, this disease remains largely incurable. We have come to realize that induction of the immune response may lead to a cure for this disease [16–21].

T cells play a central role in cell immunity. The T cell receptor (TCR) signaling cascade is initiated by engagement of antigenic peptides bound to the major histocompatibility complex (MHC). Upon TCR stimulation, CD4/CD8-associated Lck is brought into proximity with TCR/CD3 complexes and phosphorylates the immunoreceptor tyrosine-based activation motifs (ITAMs) of the CD3 subunits. ZAP70 is then recruited to the phosphorylated ITAMs and subsequently activated by Lck. Activated ZAP70 phosphorylates LAT, which in turn phosphorylates PLC*γ*1 and other signaling molecules, resulting in the activation of multiple pathways. These sequential molecular events eventually lead to T cell activation and IL-2 production.

Although the mechanism of imatinib-mediated TCR signaling inhibition has been well defined, a feasible solution for imatinib-mediated immunosuppression is unknown. As the critical tyrosine kinase in the TCR signaling pathway, Lck is vital for T cell activation. In Lck-deficient T cell lines, T cells fail to induce ZAP70 phosphorylation and Ca^+^ mobilization following TCR stimulation. On this basis, Lck inhibition by imatinib due to its sequence homology with ABL kinase in the adenosine triphosphate (ATP) binding pocket consequentially results in decreased TCR-mediated T cell activation [22]. It has been demonstrated that Lck determines the threshold for T cell activation and a lack of Lck in T cells leads to an increased activation threshold [23–25].

Because Lck expression affects T cell activation and proliferation after TCR engagement, it is reasonable to hypothesize that the Lck upregulated during imatinib treatment decreases the threshold of T cell activation and to some extent may enhance T cell activation, proliferation, and cytokine production. Unlike conventional peptide antigens, bacterial superantigens (SAgs) are protein toxins that bind to the external surfaces of the TCR and MHC class II to simultaneously activate large numbers of T cells [26]. Therefore, SAgs have been considered to activate T cells through the canonical signaling pathway, which includes TCR engagement of peptide-MHC complexes. Previous findings have shown that the superantigen staphylococcal enterotoxin A (SEA) not only powerfully activates T cells, but also induces broad expression of genes related to cytokine production and TCR signal transduction [27–29], implying that SEA may affect the expression of early kinases in the TCR signaling pathway. The aim of this study was to investigate the effect of SEA on the expression of Lck, Fyn, and ZAP70 and determine whether this effect could antagonize the imatinib-mediated inhibition of T cell activation. We demonstrate that SEA upregulates the expression and phosphorylation of Lck and subsequently avoids the imatinib-mediated inhibition of T cell activation.

## 2. Materials and Methods

### 2.1. Cell Preparation, Chemicals, and Antibodies

The Jurkat cell line was obtained from American Type Culture Collection (ATCC). Peripheral blood mononuclear cells (PBMCs) were obtained from four healthy donors who provided informed consent. Mononuclear cells were isolated by Ficoll-diatrizoate density gradient centrifugation (Sigma Chemical Co., USA). Imatinib mesylate (Glivec, STI571) was purchased from Selleck Chemicals (USA), and SEA was obtained from Sigma (USA). The following antibodies were used as primary antibodies : rabbit monoclonal anti-Lck, anti-Fyn, and anti-ZAP70 (Epitomics, USA), mouse monoclonal anti-GAPDH (Santa Cruz Biotechnology, USA), mouse monoclonal anti-phospho-Lck (Tyr394) (GeneTex, USA), rabbit monoclonal anti-phospho-ZAP70 (Tyr319) (Cell Signaling Technology, USA), and rabbit monoclonal anti-phospho-PLC*γ*1 (Tyr783) (Epitomics, USA). Secondary antibodies were purchased from Santa Cruz Biotechnology (USA).

### 2.2. Cell Culture

Jurkat cells were maintained in RPMI 1640 medium (Gibco, USA) supplemented with 10% fetal bovine serum (Hangzhou Sijiqing Company, China), 100 U/mL penicillin, and 100 *μ*g/mL streptomycin (Invitrogen) at 37°C in a humidified incubator with 5% CO_2_. Culture media was passaged every 2-3 days.

### 2.3. ELISA for IL-2

Jurkat cells or human PBMCs (1 × 10^5^/well) in a total volume of 200 *μ*L were incubated in 96-well plates in the presence or absence of SEA (20 ng/mL) for 24 h and subsequently washed with PBS. Afterward, cells were treated with or without imatinib (40 nM) for 1 h followed by stimulation with anti-CD3/CD28 coated beads at a cell : bead ratio of 5 : 1 for 24 h. The IL-2 content in the supernatant was measured with an ELISA kit (RayBiotech, USA) following the manufacturer's protocol.

### 2.4. Cell Proliferation Assay

Cells were treated the same as above. After treatment, 10 *μ*L of CCK-8 solution (Dojindo, Japan) was added into each well, and the cells were incubated at 37°C for 2 h. The sample absorbance was then recorded at 450 nm.

### 2.5. Western Blotting Analysis

Jurkat cells (3 × 10^6^/well) were cultured in 6-well plates with different concentrations of SEA for 24 h. Cells without treatment served as a negative control. The expression of Lck, Fyn, and ZAP70 was examined by Western blotting [7]. Briefly, cells were lysed in 1 mL of lysis buffer containing 1% Triton X-100. Protein concentrations were determined by the Bradford assay. The proteins were separated by electrophoresis in a 10% SDS-polyacrylamide gel and transferred onto a PVDF membrane. The blots were blocked with 5% (w/v) nonfat dry milk constituted in 1x TBS-T for 1 h at room temperature. Membranes were incubated at 4°C overnight with a primary monoclonal antibody in 5% bovine serum albumin (BSA) in 1x TBS-T and then with the respective horseradish peroxidase-conjugated secondary antibody as directed by the manufacturer for 1 hour at room temperature. Immunoreactive bands were visualized using the enhanced chemiluminescence light (ECL) detection reagent.

To assess the effects of SEA on the imatinib-mediated inhibition of the TCR signaling pathway, Jurkat cells and PBMCs (3 × 10^6^/well) were pretreated with and without SEA (20 ng/mL) for 24 h followed by imatinib or control stimulation (40 nM) for 1 hour. Finally, cells were stimulated with anti-CD3 and anti-CD28 coated Dynabeads (Dynal, Invitrogen, Oslo, Norway) at a cell :  bead ratio of 1 : 1 for 15 min [28]. Whole-cell lysates were subjected to Western blot analysis to examine the phosphorylation level of Lck, ZAP70, and PLC*γ*1 with the respective horseradish peroxidase-conjugated secondary antibody as directed by the manufacturer.

### 2.6. Statistical Analysis

The data were analyzed using SPSS version 16.0 statistical software. Error bars indicate SDs from the mean of at least three replicates. Statistical tests were conducted using the Student's test or analysis of variance (ANOVA) where indicated.

## 3. Results

### 3.1. SEA Reduces the Inhibitory Effects of Imatinib on T Cell Proliferation and IL-2 Production

**Figure 1:**
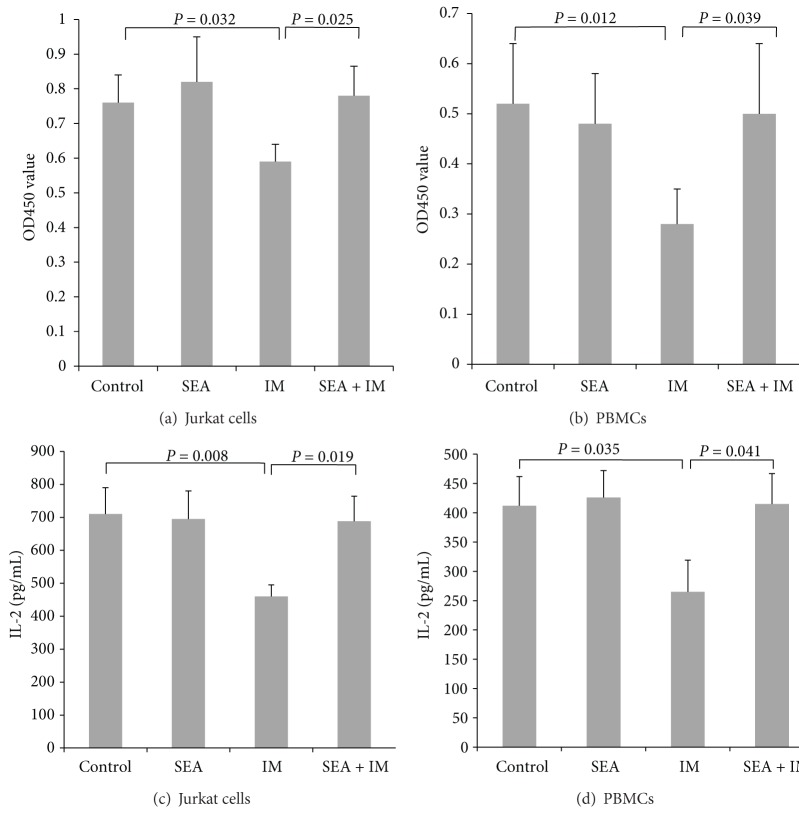
The cell proliferation and IL-2 production of Jurkat cells and PBMCs following TCR stimulation. (Control) Untreated Jurkat cells or PBMCs. (SEA) Cells were pretreated with SEA (20 ng/mL). (IM) Cells were pretreated with 40 nM imatinib. (SEA + IM) Cells were pretreated with SEA (20 ng/mL) for 24 h followed by 40 nM imatinib treatment for 15 min. Each group was stimulated with anti-CD3/CD28 coated beads at a cell : bead ratio of 5 : 1 for 24 h. Cell proliferation was assayed with the CCK-8 kit (a) and (b). The IL-2 level was assayed using a human IL-2 ELISA kit (c) and (d). The mean value and standard deviation of 3 independent experiments are shown.

It is known that imatinib suppresses T cell activation and proliferation. To investigate the effects of SEA on the inhibitory effects of imatinib on T cell proliferation and IL-2 production, Jurkat cells and PBMCs were preincubated with SEA and then washed followed by treatment with imatinib and stimulation with anti-CD3/CD28 coated beads. T cell proliferation and IL-2 production were measured using the CCK-8 kit and an IL-2 ELISA kit. The T cell proliferation and the IL-2 level were significantly decreased with imatinib treatment alone, but this suppression could be reversed with SEA pretreatment (*P* < 0.05) (Figure 1).

### 3.2. SEA Upregulates Lck and ZAP70 in a Dose-Dependent Manner

The effects of SEA on the imatinib-mediated inhibition of T cell activation and proliferation could be due to the upregulation of proximal signaling events mediated by the TCR because imatinib could inhibit TCR signal transduction [7]. Therefore, we used the well-characterized Jurkat T cell line to determine the level of signaling protein expression in response to SEA.

**Figure 2:**
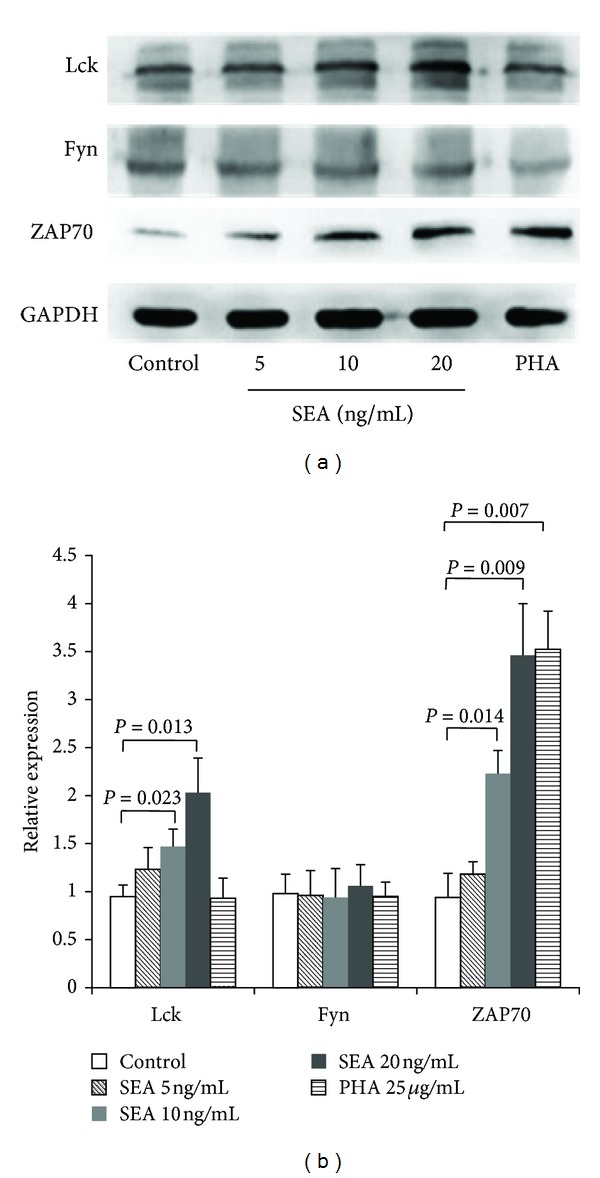
SEA increases Lck and ZAP70 expression in Jurkat cells. (a) Western blot analysis of Lck, Fyn, and ZAP70 after 24 h of incubation with the indicated concentrations of SEA. GAPDH served as a loading control. (b) Densitometry (target protein : GAPDH ratio normalized to control) was conducted on three independent experiments and is depicted in the bar graph.

Jurkat cells were incubated with different concentrations of SEA ranging from 5 to 20 ng/mL for 24 h, and cell lysates were subjected to Western blot analysis. The results demonstrated that SEA increases Lck and ZAP70 protein expression in a dose-dependent manner, but there was no effect on Fyn expression (Figure 2).

### 3.3. SEA Abrogates the Inhibitory Effects of Imatinib on TCR Signaling

**Figure 3:**
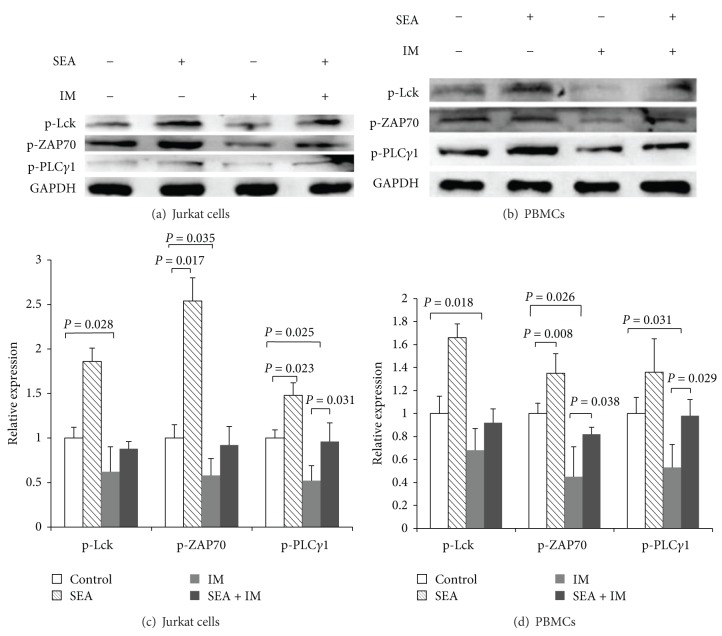
SEA pretreatment inhibited the reduction in imatinib-induced phosphorylation of Lck, ZAP70, and PLC*γ*1 in Jurkat cells (a) and PBMCs (b). Densitometry (target protein : GAPDH ratio normalized to control) was conducted on three independent experiments in Jurkat cells (c) and PBMCs (d). Jurkat cells or PBMCs were pretreated in the absence or presence of SEA (20 ng/mL) followed by treatment with or without imatinib (40 nM), and cells were then stimulated with anti-CD3/CD28 coated beads for 15 min. Total cell extracts were resolved by SDS-PAGE and subjected to immunoblot analysis using phosphospecific antibodies as indicated.

It has been reported that imatinib selectively inhibits Lck and its downstream signaling molecules [7]. Given the restricted expression of Lck, which binds to CD4/8 and is located within the vicinity of TCR-CD3 complex, as a superantigen, SEA not only causes upregulation of the Lck and ZAP70 proteins but also might cause an increase in the phosphorylation of Lck and downstream proteins by binding the TCR V beta chain. We asked whether the upregulation of Lck by SEA could prevent the suppressive effects of imatinib during T cell activation. To test this hypothesis, we measured the phosphorylation of Lck and its downstream molecules during T cell stimulation. We found that the Lck, ZAP70, and PLC*γ*1 phosphorylation in T cells, including the Jurkat cell line and PBMCs, could be increased by SEA and the reduction in the activating phosphorylations of these four signaling proteins caused by imatinib was prevented after pretreatment with SEA followed by TCR stimulation (Figure 3). SEA rescues T cells from inhibition by imatinib.

## 4. Discussion

Small molecule tyrosine kinase inhibitors have been successfully used for the treatment of CML. Imatinib and dasatinib are two of the most commonly used tyrosine kinase inhibitors, and both have been shown to impact T cell function. Imatinib inhibits T cell receptor-mediated T cell activation and proliferation in a dose-dependent manner [1, 7]. Brauer et al. described the downregulation of several LAAs in CMLs treated with imatinib [30]. Due to this activity, their use as potential immunosuppressants has been proposed. These agents have been recently used to treat autoimmune diseases, such as immune-mediated kidney injury and rheumatoid arthritis. Tyrosine kinase inhibitors (TKIs) are effective for the treatment of these diseases [31, 32].

With the development and use of TKIs being rapidly expanding, their potential side effects against cancer are of considerable clinical importance. The T cell suppressive effects of imatinib and dasatinib have been attributed to Lck inhibition. Our study is consistent with published results demonstrating that imatinib perturbs TCR signaling, inhibiting the phosphorylation of LCK and Zap70 following TCR stimulation (Figure 3).

Lck, a src family kinase, is critical for T cell activation, and its activity is tightly regulated in lipid rafts. Indeed, an appropriate balance between active and inactive Lck is essential for safe TCR sensitivity, and abnormal T cell responsiveness occurs if the balance is broken. On one hand, an absence or reduction in Lck activity leads to an impairment in the activation of mature T cells. On the other hand, increased Lck phosphorylation is associated with T cell hyperresponsiveness, which can induce autoimmune disease such as that exhibited by SLE patients [33, 34]. With respect to the role of Lck in T cell activation, Lovatt et al. support the suggestion that Lck sets a threshold of activation in T cells [24]. Accordingly, Lck is undoubtedly a key protein in the strategy of eliminating imatinib-induced immunosuppression.

Effective biologic treatment is a promising solution for tumors. In particular, the existence of a suppressed immune response directed against tumors can be relieved by treatment, or, alternatively, treatment needs to be combined with an immune-enhancing therapy. In general, bacterial SAgs can simultaneously interact with MHC II and TCR molecules to activate T cells, which produce cytokines such as IL-2 and TNF-*α* and both directly and indirectly enhance immunotherapy efficiency [35–37]. However, there were reports that superantigens can mediate T-cell-dependent killing of tumor cells independently of MHC class II molecule expression on the target cell and the Asp227Ala replacement was introduced to destroy the site having the highest affinity for MHC class II in SEA to decrease the reactivity of the superantigen with MHC class II bearing cells for the treatment of human colorectal cancer [38–40]. These studies indicated that a direct superantigen-TCR interaction could result in superantigens activation of T cells in a MHC class II-free system. And these findings could support our results that SEA could directly stimulate Jurkat cells.

Bacterial SAgs cause polyclonal T cell activation due to their capacity to bind different TCR V*β* chains [41–43]. Jurkat cells are originally from TCR V*β*8 T cell clone. And the V*β*8 chain could react with SEA weakly [44]. Lck is activated after TCR engagement with SAgs [45]. Dauwalder et al. studied the early kinetics of the transcriptional response of PBMCs primed with SEA and SEG. These authors concluded that SEA, in contrast with SEG, induced the early transcriptional activation of several pathways, including T cell activation mediators and TCR-mediated signaling [29]. Although several reports have shown that bacterial SAgs can activate human T cells lacking Lck, suggesting the existence of an additional TCR signaling pathway [46, 47], in this study, we demonstrated that SEA could upregulate Lck and ZAP70 expression and phosphorylation following TCR stimulation. SEA has shown effects that are antagonistic to the imatinib-mediated inhibition of Lck phosphorylation and downstream signaling molecules after T cells were pretreated with SEA (Figure 3). This antagonistic effect may be due to an increase in Lck and ZAP70 expression. Because the imatinib-induced inhibition of Lck phosphorylation occurs in a dose-dependent manner [7], the amount of phosphorylated Lck reduction is limited at a certain concentration of imatinib. Thus, Lck is able to trigger TCR-mediated expansion signals if basal Lck expression is increased prior to imatinib treatment.

In contrast with the conventional paradigm for T cell activation in which Lck plays a critical activating role in the TCR signaling process, Criado and Madrenas reported that Lck is dispensable for T cell activation by SAgs, but it actively inhibits this signaling pathway. The disruption of Lck function led to increased IL-2 production in response to SAgs stimulation [48]. These observations could be explained by the multistage role of Lck in T cell activation signaling based on the two pools of Lck detected at the immunological synapse, that is, the central pool and the peripheral pool. Lck may have a dual role where, early in TCR engagement, the central Lck pool participates in the initiation and activation of downstream signaling molecules and the peripheral Lck pool downregulates TCR-dependent signaling [49].

In conclusion, although the immunomodulatory effects of imatinib and dasatinib remain controversial [50], it has been confirmed that increasing effective and specific immunotherapies involving vaccination or adoptive cellular immunotherapy are necessary for CML. In this study, we showed that imatinib may affect TCR-mediated immune responses and characterized the effects of SEA on the imatinib-mediated inhibition of T cell reactivation in which this superantigen increased the tyrosine phosphorylation of Lck, ZAP70, and PLC*γ* in T cells. This finding suggests that the SEA could be used for the prevention of imatinib-mediated T cell immunosuppression. In addition, increasing Lck upregulation is a feasible option for restimulating the immune response in CML patients with TKI treatment.
